# 1,4-Diazo­niacyclo­hexane bis­(3-carb­oxy­pyrazine-2-carboxyl­ate) dihydrate

**DOI:** 10.1107/S1600536810040109

**Published:** 2010-10-13

**Authors:** Hossein Eshtiagh-Hosseini, Nafiseh Alfi, Masoud Mirzaei, Marek Necas

**Affiliations:** a Department of Chemistry, School of Sciences, Ferdowsi University of Mashhad, Mashhad 917791436, Iran; b Department of Chemistry, Faculty of Science, Masaryk University, Kamenice 5, Brno, 625 00, Czech Republic

## Abstract

In the title compound, C_4_H_12_N_2_^2+^·2C_6_H_3_N_2_O_4_^−^·2H_2_O or (1,4-dacH_2_)(pyzdcH)_2_·2H_2_O, the complete dication is generated by crystallographic inversion symmetry. An intra­molecular O—H⋯O hydrogen bond occurs in the anion. In the crystal, O—H⋯O, O—H⋯N, N—H⋯O and N—H⋯N hydrogen bonds result in the formation of a three-dimensional network. Additionally, π–π stacking inter­actions between the pyrazine rings with centroid–centroid distances of 3.7065 (2) Å are observed.

## Related literature

For related structures dereived from pyrazine-2,3-dicarb­oxy­lic acid with various organic bases, see: Eshtiagh-Hosseini *et al.* (2010*a* ▶,*b* ▶,*c* ▶,*d* ▶). For the biological properties of derivatives of 1,4-diazo­nia-cyclo­hexane derivatives, see Iqbal *et al.* (2001 ▶), Greenberg *et al.* (1981 ▶). 
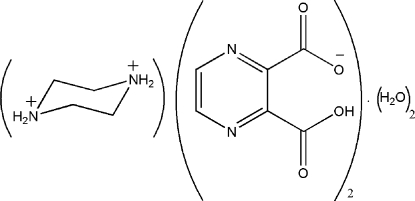


## Experimental

### 

#### Crystal data

C_4_H_12_N_2_^2+^·2C_6_H_3_N_2_O_4_^−^·2H_2_O
*M* *_r_* = 458.40
Monoclinic, 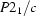

*a* = 7.7519 (4) Å
*b* = 18.4576 (8) Å
*c* = 7.0292 (4) Å
β = 111.974 (6)°
*V* = 932.68 (8) Å^3^
*Z* = 2
Mo *K*α radiation
μ = 0.14 mm^−1^
*T* = 120 K
0.40 × 0.40 × 0.30 mm

#### Data collection

Oxford Diffraction Xcalibur diffractometer with a Sapphire2 detector
Absorption correction: multi-scan (*CrysAlis RED*; Oxford Diffraction, 2009 ▶) *T*_min_ = 0.990, *T*_max_ = 1.000
4000 measured reflections
2006 independent reflections
1696 reflections with *I* > 2σ(*I*)
*R*_int_ = 0.010

#### Refinement

*R*[*F* ^2^ > 2σ(*F* ^2^)] = 0.033
*wR*(*F* ^2^) = 0.087
*S* = 1.02
2006 reflections
165 parameters
H atoms treated by a mixture of independent and constrained refinement
Δρ_max_ = 0.24 e Å^−3^
Δρ_min_ = −0.38 e Å^−3^

### 

Data collection: *CrysAlis CCD* (Oxford Diffraction, 2009 ▶); cell refinement: *CrysAlis RED* (Oxford Diffraction, 2009 ▶); data reduction: *CrysAlis RED*; program(s) used to solve structure: *SHELXS97* (Sheldrick, 2008 ▶); program(s) used to refine structure: *SHELXL97* (Sheldrick, 2008 ▶); molecular graphics: *DIAMOND* (Crystal Impact, 2009 ▶); software used to prepare material for publication: *publCIF* (Westrip, 2010 ▶).

## Supplementary Material

Crystal structure: contains datablocks I, global. DOI: 10.1107/S1600536810040109/im2230sup1.cif

Structure factors: contains datablocks I. DOI: 10.1107/S1600536810040109/im2230Isup2.hkl

Additional supplementary materials:  crystallographic information; 3D view; checkCIF report

## Figures and Tables

**Table 1:** Hydrogen-bond geometry (Å, °)

*D*—H⋯*A*	*D*—H	H⋯*A*	*D*⋯*A*	*D*—H⋯*A*
N3—H3*B*⋯O5^i^	0.92 (2)	2.01 (2)	2.800 (1)	144 (1)
N3—H3*B*⋯O4^ii^	0.92 (2)	2.46 (2)	3.061 (1)	124 (1)
N3—H3*A*⋯O2	0.92 (2)	1.97 (2)	2.763 (1)	143 (1)
N3—H3*A*⋯N1	0.92 (2)	2.34 (2)	3.107 (2)	141 (1)
O5—H5*B*⋯O4^iii^	0.85 (2)	2.25 (2)	2.923 (1)	136 (2)
O5—H5*B*⋯N2^iii^	0.85 (2)	2.34 (2)	3.107 (1)	151 (2)
O5—H5*A*⋯O2^iv^	0.95 (2)	1.90 (2)	2.841 (1)	172 (2)
O3—H1*O*⋯O1	1.13 (2)	1.29 (2)	2.414 (1)	174 (2)

Symmetry codes: (i) 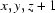; (ii) 
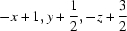
; (iii) 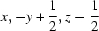; (iv) 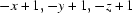.

**Fig. 1.**
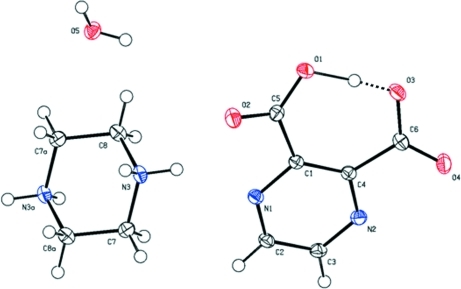
Molecular structure of the constituents of the title compound showing the atom labelling scheme. Thermal ellipsoids are presented at the 50% probability level.

**Fig. 2.**
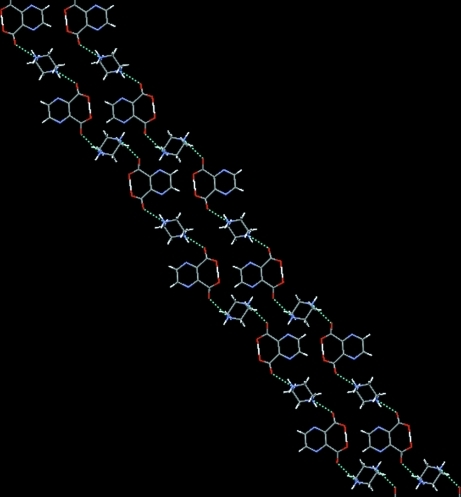
A portion of pseudo-1D polymeric chain of the title compound.

**Fig. 3.**
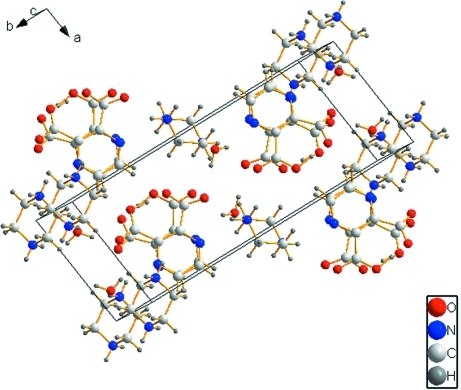
Crystal packing of the title compound.

## supplementary crystallographic information

### Comment

1,4-Dac derivatives are a broad class of chemical compounds, many with important pharmacological properties. 1,4-Dac was first introduced as an anthelmimic in 1953 to treat of common roundworms (ascariasis) and pinworms (enterobiasis; oxyuriasis) (Iqbal *et al.*, 2001; Greenberg *et al.*, 1981). The title structure reported herein contains one half of the dicationic fragment (1,4-dacH_2_)^2+^, a monoanionic fragment (pyzdcH)^-^ (pyzdcH_2_ = pyrazine-2,3-dicarboxylic acid) and one solvent water molecule per asymmetric unit (Fig. 1). The center of the 1,4-diazonia-cyclohexane dication represents a crystallographic center of inversion. The crystal structure shows that just one of the protons of pyrazine-2,3-di-carboxylic acid has been transferred to nitrogen atom of the (1,4-dacH_2_)^2+^ ring. Hydrogen bond motifs involving anionic and cationic fragments and solvent water molecules result in the formation a one dimensional chain (Fig. 2). As is obvious from the packing diagram additional π···π interactions are present in the crystal structure between adjacent pyrazine rings with centroid-centroid distances of 3.774 Å (Fig. 3).

### Experimental

The title compound was synthesized *via* the reaction between pyzdcH_2_ (0.20 g, 1.1 mmol) and 1,4-dac (0.10 g, 1.1 mmol) in a aqueous solution (10 ml) stirred for 4 h in 338 K. Slow evaporation of the solvent at r.t. yielded (1,4-dacH_2_)(pyzdcH)_2_.2H_2_O as colorless crystals after one week (yield: 30%).

### Refinement

Carbon bound hydrogen atoms were positioned geometrically and refined as riding using standard *SHELXTL* constraints, with their *U*_iso_ set to 1.2*U_eq_* of their parent atoms. Oxygen and nitrogen bound hydrogen atoms were located in a difference Fourier map and refined isotropically.

### Crystal data

**Table:**

C_4_H_12_N_2_^2+^·2C_6_H_3_N_2_O_4_^−^·2H_2_O	*F*(000) = 480
*M**_r_* = 458.40	*D*_x_ = 1.632 Mg m^−^^3^
Monoclinic, *P*2_1_/*c*	Mo *K*α radiation, λ = 0.71073 Å
Hall symbol: -P 2ybc	Cell parameters from 2589 reflections
*a* = 7.7519 (4) Å	θ = 3.0–27.5°
*b* = 18.4576 (8) Å	µ = 0.14 mm^−^^1^
*c* = 7.0292 (4) Å	*T* = 120 K
β = 111.974 (6)°	Prism, colourless
*V* = 932.68 (8) Å^3^	0.40 × 0.40 × 0.30 mm
*Z* = 2	

### Data collection

**Table:**

Oxford Diffraction Xcalibur with a Sapphire2 detector diffractometer	2006 independent reflections
Radiation source: Enhance (Mo) X-ray Source	1696 reflections with *I* > 2σ(*I*)
graphite	*R*_int_ = 0.010
Detector resolution: 8.4353 pixels mm^-1^	θ_max_ = 27.6°, θ_min_ = 3.0°
ω scan	*h* = −9→9
Absorption correction: multi-scan (*CrysAlis RED*; Oxford Diffraction, 2009)	*k* = −15→23
*T*_min_ = 0.990, *T*_max_ = 1.000	*l* = −6→8
4000 measured reflections	

### Refinement

**Table:**

Refinement on *F*^2^	Primary atom site location: structure-invariant direct methods
Least-squares matrix: full	Secondary atom site location: difference Fourier map
*R*[*F*^2^ > 2σ(*F*^2^)] = 0.033	Hydrogen site location: inferred from neighbouring sites
*wR*(*F*^2^) = 0.087	H atoms treated by a mixture of independent and constrained refinement
*S* = 1.02	*w* = 1/[σ^2^(*F*_o_^2^) + (0.0601*P*)^2^] where *P* = (*F*_o_^2^ + 2*F*_c_^2^)/3
2006 reflections	(Δ/σ)_max_ = 0.001
165 parameters	Δρ_max_ = 0.24 e Å^−^^3^
0 restraints	Δρ_min_ = −0.38 e Å^−^^3^

### Special details

**Table:**

Geometry. All e.s.d.'s (except the e.s.d. in the dihedral angle between two l.s. planes) are estimated using the full covariance matrix. The cell e.s.d.'s are taken into account individually in the estimation of e.s.d.'s in distances, angles and torsion angles; correlations between e.s.d.'s in cell parameters are only used when they are defined by crystal symmetry. An approximate (isotropic) treatment of cell e.s.d.'s is used for estimating e.s.d.'s involving l.s. planes.
Refinement. Refinement of *F*^2^ against ALL reflections. The weighted *R*-factor *wR* and goodness of fit *S* are based on *F*^2^, conventional *R*-factors *R* are based on *F*, with *F* set to zero for negative *F*^2^. The threshold expression of *F*^2^ > σ(*F*^2^) is used only for calculating *R*-factors(gt) *etc*. and is not relevant to the choice of reflections for refinement. *R*-factors based on *F*^2^ are statistically about twice as large as those based on *F*, and *R*- factors based on ALL data will be even larger.

### Fractional atomic coordinates and isotropic or equivalent isotropic displacement parameters (Å^2^)

**Table:**

	*x*	*y*	*z*	*U*_iso_*/*U*_eq_	
O1	0.66092 (11)	0.30115 (5)	0.67977 (14)	0.0214 (2)	
O2	0.47664 (11)	0.38022 (4)	0.74880 (14)	0.0213 (2)	
O3	0.70151 (11)	0.17141 (5)	0.70969 (13)	0.0198 (2)	
O4	0.54415 (12)	0.07636 (5)	0.74616 (13)	0.0219 (2)	
N1	0.19076 (13)	0.29280 (5)	0.62615 (15)	0.0149 (2)	
N2	0.22265 (13)	0.14348 (6)	0.61232 (14)	0.0156 (2)	
N3	0.15261 (14)	0.46005 (6)	0.64194 (15)	0.0157 (2)	
C1	0.35741 (15)	0.26185 (6)	0.66012 (16)	0.0126 (2)	
C2	0.04412 (16)	0.24966 (6)	0.58448 (18)	0.0159 (3)	
H2	−0.0743	0.2704	0.5607	0.019*	
C3	0.05966 (16)	0.17478 (7)	0.57477 (18)	0.0162 (3)	
H3	−0.0487	0.1456	0.5406	0.019*	
C4	0.37356 (15)	0.18606 (6)	0.65666 (17)	0.0133 (3)	
C5	0.51081 (16)	0.31916 (7)	0.70094 (17)	0.0151 (3)	
C6	0.55073 (16)	0.14025 (7)	0.70888 (17)	0.0160 (3)	
C7	−0.04171 (16)	0.44630 (7)	0.62525 (19)	0.0190 (3)	
H7A	−0.0416	0.4307	0.7600	0.023*	
H7B	−0.0968	0.4069	0.5252	0.023*	
C8	0.15674 (16)	0.48597 (7)	0.44316 (18)	0.0176 (3)	
H8A	0.1073	0.4478	0.3378	0.021*	
H8B	0.2868	0.4961	0.4588	0.021*	
O5	0.23627 (12)	0.52146 (5)	0.03053 (14)	0.0190 (2)	
H3B	0.202 (2)	0.4944 (9)	0.742 (2)	0.027 (4)*	
H3A	0.222 (2)	0.4184 (9)	0.675 (2)	0.032 (4)*	
H5B	0.271 (2)	0.4805 (10)	0.089 (3)	0.045 (5)*	
H5A	0.330 (3)	0.5564 (11)	0.092 (3)	0.071 (6)*	
H1O	0.683 (3)	0.2317 (12)	0.687 (3)	0.070 (7)*	

### Atomic displacement parameters (Å^2^)

**Table:**

	*U*^11^	*U*^22^	*U*^33^	*U*^12^	*U*^13^	*U*^23^
O1	0.0147 (4)	0.0176 (5)	0.0344 (5)	−0.0009 (4)	0.0120 (4)	0.0005 (4)
O2	0.0144 (4)	0.0146 (5)	0.0303 (5)	−0.0010 (4)	0.0032 (4)	−0.0039 (4)
O3	0.0130 (4)	0.0185 (5)	0.0284 (5)	0.0008 (4)	0.0083 (4)	−0.0032 (4)
O4	0.0200 (5)	0.0152 (5)	0.0284 (5)	0.0037 (4)	0.0067 (4)	0.0011 (4)
N1	0.0132 (5)	0.0164 (5)	0.0153 (5)	0.0006 (4)	0.0054 (4)	0.0004 (4)
N2	0.0157 (5)	0.0156 (5)	0.0164 (5)	−0.0010 (4)	0.0070 (4)	−0.0007 (4)
N3	0.0141 (5)	0.0145 (5)	0.0166 (5)	0.0030 (4)	0.0034 (4)	−0.0006 (4)
C1	0.0123 (6)	0.0155 (6)	0.0100 (5)	0.0005 (5)	0.0041 (4)	0.0002 (4)
C2	0.0117 (6)	0.0189 (6)	0.0173 (6)	0.0016 (5)	0.0056 (4)	0.0017 (5)
C3	0.0131 (6)	0.0181 (6)	0.0176 (6)	−0.0023 (5)	0.0060 (4)	0.0002 (5)
C4	0.0138 (6)	0.0158 (6)	0.0108 (5)	0.0005 (5)	0.0052 (4)	−0.0002 (4)
C5	0.0132 (6)	0.0150 (6)	0.0144 (6)	−0.0005 (5)	0.0022 (4)	0.0012 (5)
C6	0.0150 (6)	0.0168 (6)	0.0149 (6)	0.0007 (5)	0.0042 (4)	−0.0041 (4)
C7	0.0174 (6)	0.0179 (6)	0.0222 (6)	−0.0003 (5)	0.0080 (5)	0.0037 (5)
C8	0.0158 (6)	0.0208 (6)	0.0162 (6)	0.0024 (5)	0.0060 (5)	−0.0009 (5)
O5	0.0186 (5)	0.0156 (5)	0.0215 (5)	−0.0016 (4)	0.0059 (4)	0.0002 (4)

### Geometric parameters (Å, °)

**Table:**

O1—C5	1.2713 (14)	C1—C4	1.4054 (16)
O1—H1O	1.29 (2)	C1—C5	1.5362 (16)
O2—C5	1.2330 (14)	C2—C3	1.3912 (16)
O3—C6	1.3007 (14)	C2—H2	0.9500
O3—H1O	1.13 (2)	C3—H3	0.9500
O4—C6	1.2133 (15)	C4—C6	1.5359 (16)
N1—C2	1.3277 (15)	C7—C8^i^	1.5061 (17)
N1—C1	1.3496 (14)	C7—H7A	0.9900
N2—C3	1.3230 (15)	C7—H7B	0.9900
N2—C4	1.3455 (14)	C8—C7^i^	1.5061 (17)
N3—C7	1.4883 (15)	C8—H8A	0.9900
N3—C8	1.4886 (15)	C8—H8B	0.9900
N3—H3B	0.915 (16)	O5—H5B	0.852 (19)
N3—H3A	0.917 (17)	O5—H5A	0.95 (2)
			
C5—O1—H1O	111.5 (8)	C1—C4—C6	128.41 (10)
C6—O3—H1O	111.6 (10)	O2—C5—O1	124.78 (11)
C2—N1—C1	117.97 (10)	O2—C5—C1	116.74 (10)
C3—N2—C4	118.28 (10)	O1—C5—C1	118.47 (10)
C7—N3—C8	110.95 (9)	O4—C6—O3	122.63 (11)
C7—N3—H3B	107.4 (9)	O4—C6—C4	118.73 (10)
C8—N3—H3B	110.3 (9)	O3—C6—C4	118.64 (10)
C7—N3—H3A	110.9 (9)	N3—C7—C8^i^	110.15 (10)
C8—N3—H3A	107.0 (9)	N3—C7—H7A	109.6
H3B—N3—H3A	110.4 (14)	C8^i^—C7—H7A	109.6
N1—C1—C4	120.25 (10)	N3—C7—H7B	109.6
N1—C1—C5	111.37 (10)	C8^i^—C7—H7B	109.6
C4—C1—C5	128.37 (10)	H7A—C7—H7B	108.1
N1—C2—C3	121.59 (11)	N3—C8—C7^i^	110.39 (9)
N1—C2—H2	119.2	N3—C8—H8A	109.6
C3—C2—H2	119.2	C7^i^—C8—H8A	109.6
N2—C3—C2	121.18 (11)	N3—C8—H8B	109.6
N2—C3—H3	119.4	C7^i^—C8—H8B	109.6
C2—C3—H3	119.4	H8A—C8—H8B	108.1
N2—C4—C1	120.67 (10)	H5B—O5—H5A	109.8 (17)
N2—C4—C6	110.85 (10)		

Symmetry codes: (i) −*x*, −*y*+1, −*z*+1.

### Hydrogen-bond geometry (Å, °)

**Table:**

*D*—H···*A*	*D*—H	H···*A*	*D*···*A*	*D*—H···*A*
N3—H3B···O5^ii^	0.92 (2)	2.01 (2)	2.800 (1)	144 (1)
N3—H3B···O4^iii^	0.92 (2)	2.46 (2)	3.061 (1)	124 (1)
N3—H3A···O2	0.92 (2)	1.97 (2)	2.763 (1)	143 (1)
N3—H3A···N1	0.92 (2)	2.34 (2)	3.107 (2)	141 (1)
O5—H5B···O4^iv^	0.85 (2)	2.25 (2)	2.923 (1)	136 (2)
O5—H5B···N2^iv^	0.85 (2)	2.34 (2)	3.107 (1)	151 (2)
O5—H5A···O2^v^	0.95 (2)	1.90 (2)	2.841 (1)	172 (2)
O3—H1O···O1	1.13 (2)	1.29 (2)	2.414 (1)	174 (2)

Symmetry codes: (ii) *x*, *y*, *z*+1; (iii) −*x*+1, *y*+1/2, −*z*+3/2; (iv) *x*, −*y*+1/2, *z*−1/2; (v) −*x*+1, −*y*+1, −*z*+1.
